# Predictive Effect of GDF-15 on Adverse Outcomes After Cardiovascular Interventions: A Systematic Review and Meta-Analysis

**DOI:** 10.31083/RCM28279

**Published:** 2025-04-16

**Authors:** Xiaotong Jia, Junwei Gao, Zeyou Qi, Jun Ma

**Affiliations:** ^1^Department of Anesthesiology, Beijing Anzhen Hospital, Capital Medical University, 100029 Beijing, China

**Keywords:** growth differentiation factor 15, cardiac surgical procedures, acute kidney injury, mortality, atrial fibrillation

## Abstract

**Background:**

This systematic review and meta-analysis aimed to evaluate the predictive effect of Growth Differentiation Factor-15 (GDF-15) on adverse outcomes in patients undergoing cardiovascular interventions.

**Method:**

A comprehensive literature search was performed across PubMed, EMBASE, Cochrane Library, and Web of Science databases. The meta-analysis used hazard ratios (HR) and odds ratios (OR) to compare outcomes such as all-cause mortality, cardiovascular death, postoperative atrial fibrillation (AF), acute kidney injury (AKI), and spontaneous myocardial infarction (MI) between high GDF-15 levels and control groups. Subgroup analyses were conducted based on study design and GDF-15 cutoff levels. Publication bias was evaluated using funnel plot and Egger’s test.

**Results:**

A total of 13 studies were included in the meta-analysis. The study revealed a significant association between elevated GDF-15 levels and increased all-cause mortality. Subgroup analysis showed a significant association in retrospective studies but not in prospective studies. Higher GDF-15 cutoff levels (>2 ng/mL) were more strongly associated with increased mortality than lower cutoff levels (≤2 ng/mL). Elevated GDF-15 levels were found to be significantly associated with increased risks of cardiovascular death, AKI, and spontaneous MI. No significant difference was observed in the incidence of postoperative AF. The overall adverse outcomes analysis showed no significant difference. Subgroup analyses suggested significant associations primarily observed in studies with higher GDF-15 cutoffs.

**Conclusion:**

Elevated GDF-15 levels are associated with increased risks of all-cause mortality, cardiovascular death, AKI, and spontaneous MI in patients undergoing cardiovascular interventions. Due to the heterogeneity of the studies, including variations in surgical techniques, the conclusions should be interpreted with caution.

**The PROSPERO Registration:**

CRD42024582279, https://www.crd.york.ac.uk/PROSPERO/view/CRD42024582279.

## 1. Introduction

Cardiac surgeries are essential for treating a variety of heart diseases, including coronary artery disease and congenital heart defects [1, 2]. However, these procedures are associated with several potential complications, such as cardiovascular death, atrial fibrillation (AF), acute kidney injury (AKI), and spontaneous myocardial infarction (MI). AF represents a common complication, affecting 30% to 50% of patients following cardiovascular interventions [3]. The development of AF is associated with increased morbidity and mortality, including higher risks of stroke, heart failure, and prolonged hospital stays [4]. AKI occurs in a significant portion of patients undergoing cardiovascular interventions, resulting in increased morbidity and mortality [5]. The development of AKI post-surgery necessitates prolonged hospital stays and intensive care, significantly impacting patient quality of life and increasing healthcare costs [6]. Given the significant morbidity and mortality associated with these complications, it is crucial to identify patients who are at high risk to take appropriate measures, including monitoring of renal function, maintaining adequate hydration, and so on [7, 8]. Therefore, early detection and intervention are essential in mitigating potential complications.

Growth differentiation factor-15 (GDF-15), a stress-responsive cytokine, is elevated in response to myocardial stretch, inflammation, and oxidative stress, making it a potential indicator of various complications following cardiac procedures [9]. A study has explored the predictive effect of GDF-15 on adverse outcomes after cardiovascular interventions, highlighting its potential as a significant biomarker for risk stratification and outcome prediction. A prospective, single-center study found that low preoperative plasma levels of GDF-15 are a strong independent predictor of postoperative AF in patients undergoing off-pump and on-pump coronary artery bypass graft (CABG) surgery, adding predictive value to classic risk factors [10]. Kato *et al*. [11] reported preoperative levels of GDF-15 can help identify short-term operative risks including AKI, as well as 30-day mortality and morbidity in patients undergoing cardiovascular surgery. Preoperative biomarkers reflecting cardiac, inflammatory, renal, and metabolic disorders are strongly associated with cardiac surgery-associated AKI and can enhance the identification of at-risk elderly patients compared to clinical risk factors alone [12]. In revascularized patients with non-ST-elevation acute coronary syndrome, biomarkers N-terminal pro-B-type natriuretic peptide (NT-proBNP) and GDF-15 could improve the prognostication of cardiovascular death and spontaneous MI beyond clinical risk factors alone [13]. Wollert *et al*. [14] reported that elevated GDF-15 levels improve risk stratification in non-ST-elevation acute coronary syndrome, predicting outcomes better with an invasive strategy. Given the diverse findings across multiple studies regarding the predictive value of GDF-15 on adverse outcomes after cardiovascular interventions, conducting a meta-analysis and systematic review is crucial to consolidate this evidence, resolve inconsistencies, and provide robust, comprehensive insights.

The aim of this systematic review and meta-analysis is to evaluate the predictive effect of GDF-15 on adverse outcomes following cardiovascular interventions. The hypothesis of this systematic review and meta-analysis is that elevated preoperative GDF-15 are significant predictors of adverse outcomes in patients undergoing cardiovascular interventions.

## 2. Materials and Methods

In compliance with the Preferred Reporting Items for Systematic Reviews and Meta-Analyses (PRISMA) guidelines, this systematic review and meta-analysis aimed to evaluate the prognostic value of GDF-15 for adverse outcomes following cardiovascular interventions [15]. We have registered our study on the International Prospective Register of Systematic Reviews (PROSPERO) with the registration number CRD42024582279.

### 2.1 Search Strategy

A comprehensive literature search was performed across four major databases including PubMed, EMBASE, Cochrane Library, and Web of Science up to April 1, 2024. The objective of this search was to identify studies examining the prognostic value of GDF-15 in patients undergoing cardiovascular interventions. The search strategy encompassed a broad range of terms related to GDF-15 and cardiac surgeries, ensuring the inclusion of all relevant studies without language or publication date restrictions. The search terms combined various keywords and Medical Subject Headings (MeSH) terms related to GDF-15 and cardiac surgical procedures. Specific terms for GDF-15 included “macrophage inhibitory cytokine-1 protein human”, “macrophage inhibitory cytokine-1 (MIC-1) protein human”, “GDF-15 protein human”, among other relevant variations. For cardiac surgical procedures, terms included “Cardiac Surgical Procedures”, “Heart Surgical Procedures”, and “heart surgery”. The detailed search strategies for each database are outlined in **Supplementary Table 1**.

### 2.2 Inclusion and Exclusion Criteria

The inclusion criteria for this systematic review and meta-analysis were defined as follows: (1) patients undergoing cardiovascular interventions (excluding heart transplants), (2) studies measuring GDF-15 levels, (3) studies comparing high level GDF-15 levels patients with patients with low GDF-15 levels, and (4) outcome measures including AKI, mortality, postoperative AF [16], and cardiovascular death. The exclusion criteria included: (1) reviews, conference papers, research designs, case reports, and other non-original research articles, (2) duplicate studies, and (3) studies with non-extractable data.

### 2.3 Study Selection

Eligibility of the retrieved records was assessed by two independent reviewers through the examination of titles and abstracts. Subsequently, a thorough evaluation of the full texts of potentially relevant studies was performed. Discrepancies were resolved through discussion. The selection process was documented using a PRISMA flow diagram.

### 2.4 Data Extraction and Quality Assessment

Data from each included study were extracted by two independent reviewers. The extracted data comprised study design, country of origin, patient population, sample size, events in experimental and control groups, average age of participants, percentage of female participants, type of intervention, method of GDF-15 testing, comparison groups, cut-off values for GDF-15 levels, and reported outcomes. Specifically, data on the incidence of adverse outcomes such as AKI, AF, all-cause mortality, cardiovascular death, and spontaneous MI were collected. The methodological quality of the included studies was assessed using the Newcastle-Ottawa scale (NOS), a 9-point scale evaluating studies based on selection criteria, comparability of groups, and the determination of either exposure or outcome [17]. Discrepancies encountered during data extraction or quality assessment were resolved through discussion. If consensus could not be reached, a third reviewer was consulted to resolve disagreements.

### 2.5 Data Analysis

Statistical analyses were conducted using Stata 12.0 (StataCorp, College Station, TX, USA). Count data were compared using hazard ratios (HR) or odds ratios (OR) with their corresponding 95% CIs. The choice between HR and OR depended on the type of data reported in the included studies. The selection between fixed-effects and random-effects models was determined by the observed heterogeneity among the included studies. Heterogeneity was assessed using the I^2^ statistic, with values exceeding 50% indicating substantial heterogeneity. Fixed-effects models were applied for studies with low heterogeneity (I^2^ ≤ 50%), while random-effects models were used for studies with high heterogeneity (I^2^ > 50%). Subgroup analyses were conducted to investigate the effects of various variables, such as study design and GDF-15 cut-off values, on the outcomes. Sensitivity analyses were conducted using leave-one-out method to assess the robustness of the synthesized results. Publication bias was assessed visually through funnel plot inspection and statistically using Egger’s test [18]. The funnel plot was examined for asymmetry, and Egger’s test was applied to quantify the bias. To further address publication bias, a trim-and-fill analysis was performed, which provides a more accurate estimate by accounting for potential unpublished studies.

## 3. Results

### 3.1 Study Selection

**Fig. 1.**
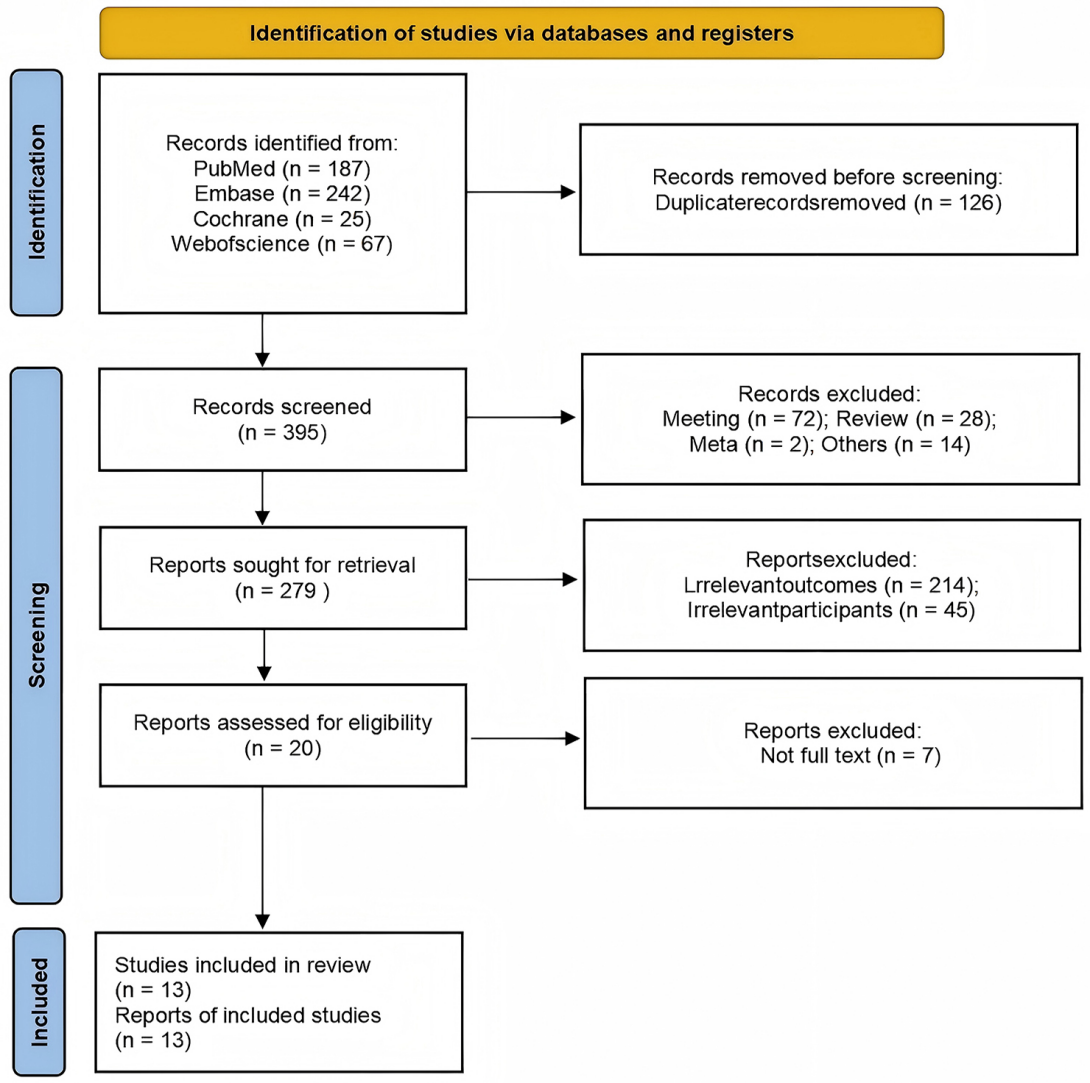
**PRISMA study selection flow diagram**. PRISMA, Preferred Reporting Items for Systematic Reviews and Meta-Analyses.

As shown in Fig. 1, a comprehensive search identified 521 records. After removing 126 duplicates, 395 studies remained for title and abstract screening. During screening, 116 records were excluded: 72 meeting abstracts, 28 review articles, 2 meta-analyses, and 14 unrelated studies. This resulted in 279 reports remaining for eligibility assessment. Upon detailed examination, 259 reports were excluded due to irrelevant outcomes (214 reports) and irrelevant participants (45 reports). Additionally, 7 reports were excluded for not being available in full text, resulting in 13 studies included in the meta-analysis.

### 3.2 Characteristics of Included Studies

The meta-analysis included 13 studies, conducted between 2007 and 2024, evaluating the prognostic effect of GDF-15 on adverse outcomes post-cardiovascular interventions (Table 1, Ref. [10, 11, 12, 13, 14, 19, 20, 21, 22, 23, 24, 25, 26]). These geographically diverse studies included research from Germany, the Netherlands, France, Sweden, Switzerland, Japan, and Canada. Of the included studies, eight were prospective, while five were retrospective. The total sample size was 21,629 patients, undergoing various cardiac procedures such as elective cardiac surgery, ST-segment elevation MI interventions, coronary artery bypass surgery, transcatheter aortic valve implantation, and AF ablation. The mean age of participants ranged from 59 to 84 years (Table 1). The proportion of female participants ranged from 8% to 55.8%, reflecting broad demographic variation. Various methods were employed for measuring GDF-15 levels, including electrochemiluminescence immunoassay (ECLIA) and enzyme-linked immunosorbent assay (ELISA), with different cut-off values to distinguish high from low GDF-15 levels. Outcome measures focused on adverse events such as AKI, all-cause mortality, postoperative AF, cardiovascular death, spontaneous MI, and sarcopenia, evaluated using OR and HR (Table 1). The quality assessment of the studies was performed using the NOS. The NOS scores for included studies ranged from 7 to 9 points, demonstrating good methodological quality (**Supplementary Table 2**).

**Table 1.** **Characteristic of the included studies**.

Study (first author)	Study design	Country	Patients	Sample size	Eg (event/total)	Cg (event/total)	Age	Female %	Intervention	Method of testing	Comparison	Cut-off	Outcome
Heringlake 2016 [22]	prospective study	Germany	after elective cardiac surgery	1176	85/392	37/392	68	31.1	cardiac surgery	ECLIA	high GDF-15 vs. low GDF-15	0.989 ng/mL	OR: AKI
Bodde 2019 [19]	retrospective study	Netherlands	ST-segment elevation myocardial infarction patients	290	28/145	9/145	59	22.4	pPCI	ECLIA	high GDF-15 vs. low GDF-15	37.8 pmol/L	HR: all-cause mortality
Verwijmeren 2021 [12]	prospective study	Netherlands	elderly patients	539	14/88	9/451	74.16	33.58	cardiac surgery	ECLIA	high GDF-15 vs. low GDF-15	2.199 ng/mL	OR: AKI
Bouchot 2015 [10]	prospective study	France	after coronary artery bypass surgery	100	6/34	5/66	64.02	8	Coronary artery bypass surgery	ELISA	high GDF-15 vs. low GDF-15	1.013 ng/mL	OR: AF
Lindholm 2017 [13]	retrospective study	Sweden	Non–ST-elevation acute coronary syndrome	5174	59/1293	8/1293	63	25	PCI or coronary artery bypass graft	ECLIA	high GDF-15 vs. low GDF-15	2.052 ng/mL	HR: cardiovascular death, spontaneous MI
El-Harasis 2024 [20]	prospective study	Switzerland	after AF ablation	1873	\	\	66.1	36.5	AF ablation	ECLIA	high GDF-15 vs. low GDF-15	\	OR: AF
Guenancia 2015 [21]	prospective study	France	after cardiac bypass surgery	134	\	\	64.88	11.19	cardiac bypass surgery	ELISA	high GDF-15 vs. low GDF-15	1.033 ng/mL	OR: AKI
Kato 2021 [11]	prospective study	Japan	after cardiovascular surgery	145	\	\	68.4	36.55	cardiovascular surgery with cardiopulmonary bypass	ELISA	high GDF-15 vs. low GDF-15	1.851 ng/mL	OR: AKI
Krau 2015 [24]	prospective study	Germany	after transcatheter aortic valve implantation	217	29/54	37/163	81.8	55.8	transcatheter aortic valve implantation	ELISA	high GDF-15 vs. low GDF-15	2.256 ng/mL	HR: all-cause mortality
Kim 2017 [23]	retrospective study	Canada	after transcatheter aortic valve replacement	112	\	\	84	34	Transcatheter aortic valve replacement	ELISA	high GDF-15 vs. low GDF-15	2 ng/mL	HR: all-cause mortality
Sinning 2015 [25]	prospective study	Germany	after transcatheter aortic valve replacement	310	\	\	82	46.6	transcatheter aortic valve replacement	ECLIA	high GDF-15 vs. low GDF-15	2.567 ng/mL	HR: all-cause mortality
Velders 2015 [26]	retrospective study	Sweden	ST-segment elevation myocardial infarction patients	5385	118/1346	18/1346	59	22.6	pPCI	ELISA	high GDF-15 vs. low GDF-15	1.116 ng/mL	HR: cardiovascular death, spontaneous MI
Wollert 2007 [14]	prospective study	Sweden	Non–ST-elevation acute coronary syndrome	2079	37/253	40/416	66	30.3	coronary artery bypass graft	ELISA	high GDF-15 vs. low GDF-15	1.2 ng/mL	HR: cardiovascular death, spontaneous MI

ECLIA, electrochemiluminescence immunoassay; ELISA, enzyme-linked immunosorbent assay; GDF-15, growth differentiation factor-15; OR, odds ratios; HR, hazard ratios; AKI, acute kidney injury; AF, atrial fibrillation; MI, myocardial infarction; Eg, experimental group; Cg, control group; pPCI, primary percutaneously coronary intervention.

### 3.3 Meta-Analysis of Overall Adverse Outcomes

**Fig. 2.**
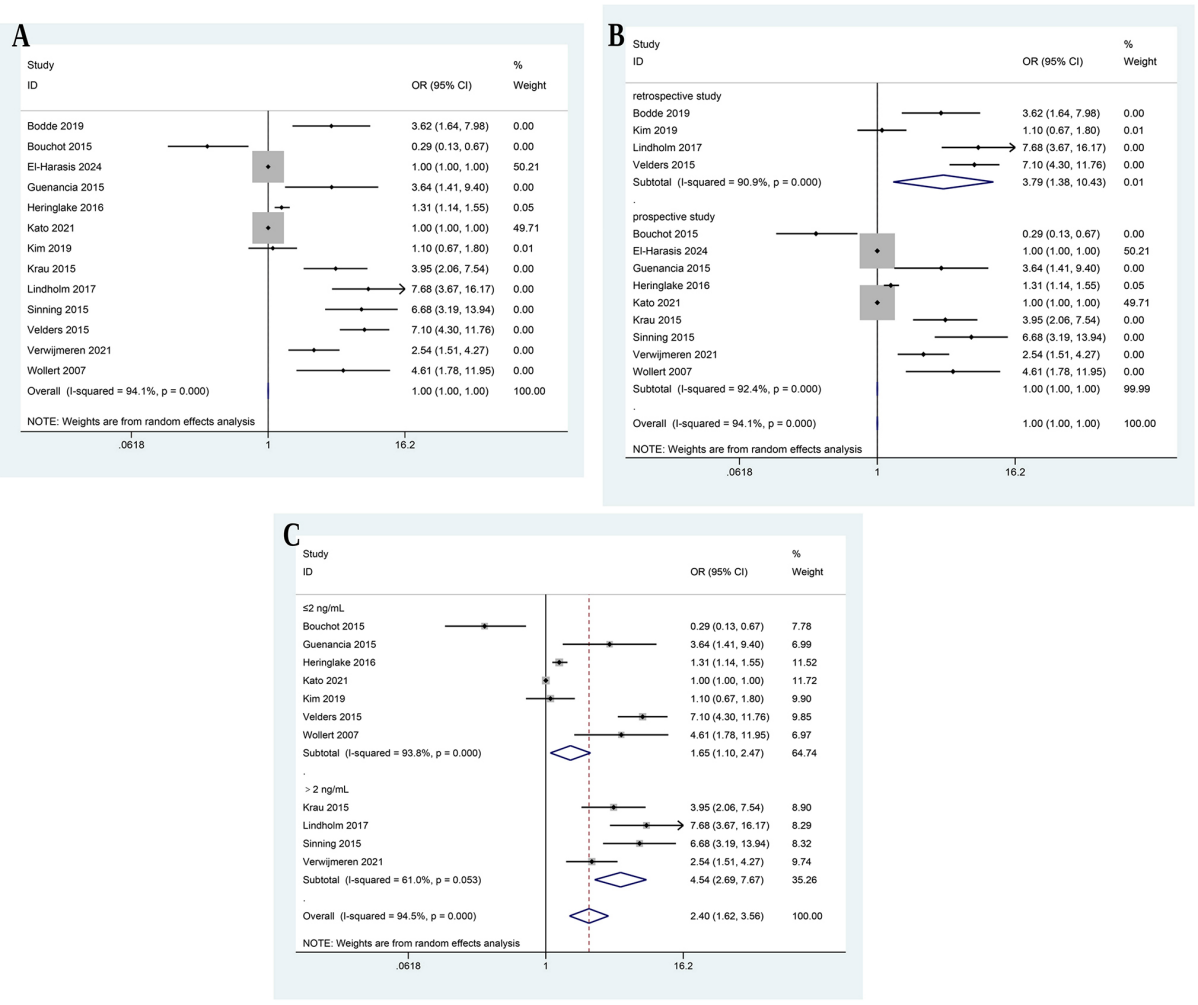
**Meta-analysis of incidence of overall adverse outcomes**. Forest plot (A), subgroup analysis by study design (B) and cut-off (C).

A total of thirteen studies were pooled for the predictive effect of GDF-15 on overall adverse outcomes in patients undergoing various cardiac surgeries [10, 11, 12, 13, 14, 19, 20, 21, 22, 23, 24, 25, 26]. The pooled OR for the incidence of overall adverse outcomes was estimated to be 1.001 (95% CI: 0.998 to 1.005, *p* = 0.575), indicating no significant difference in the incidence of overall adverse outcomes between high GDF-15 levels and control groups (Fig. 2A). The subgroup analysis by study design divided studies into two groups: retrospective and prospective. For the retrospective subgroup, the pooled OR for overall adverse outcomes was estimated to be 3.793 (95% CI: 1.379 to 10.429, *p* = 0.010), indicating a significant association between high GDF-15 levels and overall adverse outcomes (Fig. 2B). For the prospective subgroup, the pooled OR for overall adverse outcomes was estimated to be 1.001 (95% CI: 0.998 to 1.003, *p* = 0.589), indicating no significant association between high GDF-15 levels and overall adverse outcomes (Fig. 2B). The subgroup analysis by cut-off levels divided studies into two groups: those with a GDF-15 cut-off of ≤2 ng/mL and those with a cut-off of >2 ng/mL. For the ≤2 ng/mL subgroup, the pooled OR for overall adverse outcomes was estimated to be 1.648 (95% CI: 1.097 to 2.475, *p* = 0.016), indicating a significant association between high GDF-15 levels and overall adverse outcomes (Fig. 2C). For the >2 ng/mL subgroup, comprising four studies, the pooled OR for overall adverse outcomes was estimated to be 4.544 (95% CI: 2.690 to 7.674, *p* = 0.000), indicating a significant association between high GDF-15 levels and overall adverse outcomes (Fig. 2C).

### 3.4 Meta-Analysis of All-Cause Mortality

**Fig. 3.**
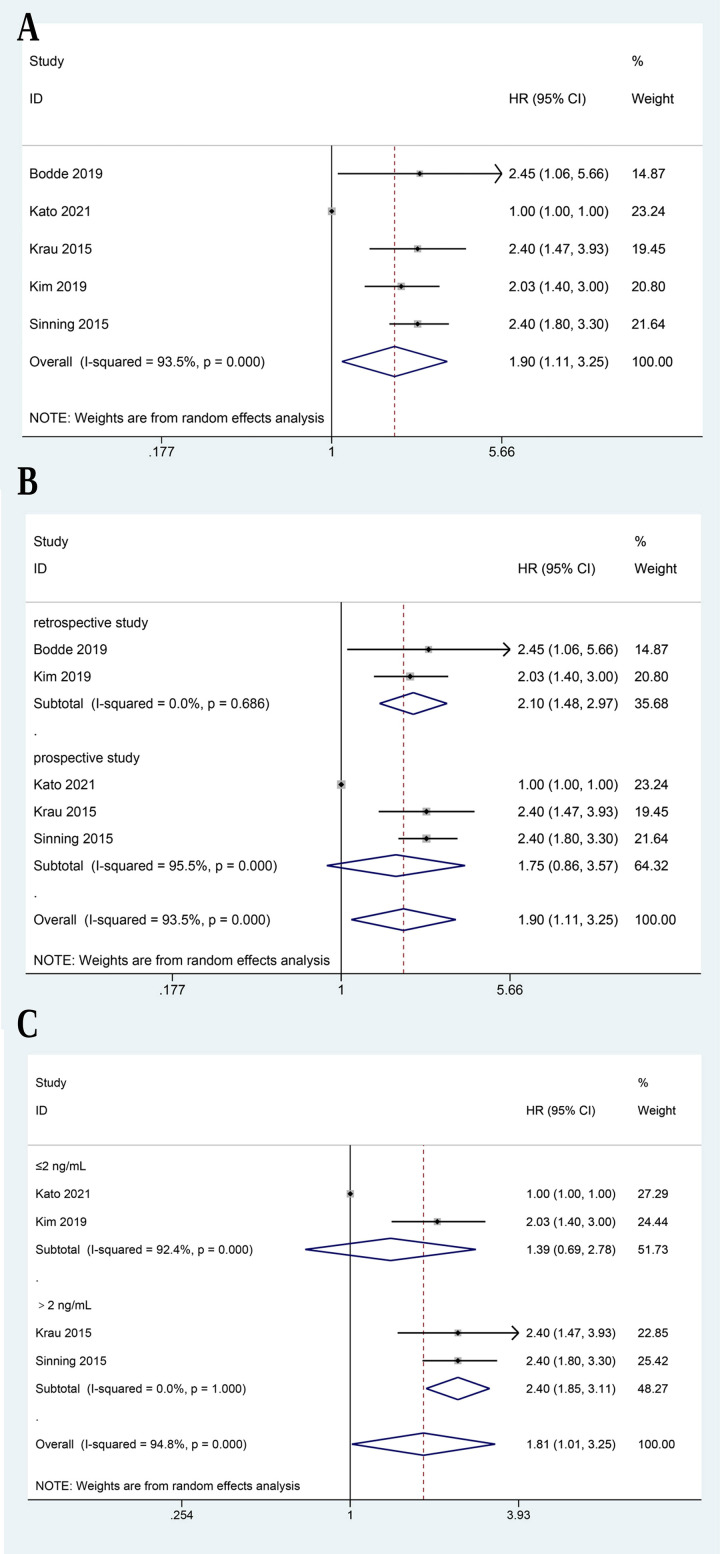
**Meta-analysis of incidence of all-cause mortality**. Forest Plot (A), subgroup analysis by study design (B) and cut-off (C).

The meta-analysis assessed the association between GDF-15 levels and all-cause mortality rates in patients undergoing various cardiac surgeries, which included data from five studies [11, 19, 23, 24, 25]. There was significant heterogeneity among the studies (I^2^ = 93.5%, *p* = 0.000), and randnom-effect model was adopted. The pooled HR for all-cause mortality was estimated to be 1.898 (95% CI: 1.109 to 3.247), indicating a significant association between elevated GDF-15 levels and increased mortality rates (*p* = 0.019, Fig. 3A). Sensitivity analysis demonstrated the robustness of the meta-analysis results (**Supplementary Fig. 1**). Subgroup analysis was conducted based on study design (retrospective vs. prospective). The retrospective subgroup included two studies [19, 23]. The pooled HR for all-cause mortality in this subgroup was 2.097 (95% CI: 1.483 to 2.966), indicating a significant association between high GDF-15 levels and increased mortality rates in the retrospective studies (*p* < 0.01, Fig. 3B). The prospective subgroup comprised three studies [11, 24, 25]. The pooled HR for all-cause mortality in this subgroup was 1.755 (95% CI: 0.863 to 3.569), indicating that it was not statistically significant between high GDF-15 levels and increased mortality rates in the prospective studies (*p* = 0.121, Fig. 3B). Furthermore, a subgroup analysis was performed based on the cutoff values of GDF-15 levels: ≤2 ng/mL and >2 ng/mL, as included studies have reported cut-off values approximately within the range of 1 to 3 ng/mL, which justifies the use of 2 ng/mL as a reference point to explore the impact of different cut-off levels on the results (Table 1). The pooled HR for all-cause mortality in subgroup of GDF-15 cutoff value of ≤2 ng/mL was 1.388 (95% CI: 0.694 to 2.778, *p* = 0.354), indicating no significant difference in mortality rates (Fig. 3C). The pooled HR for the subgroup of studies with a GDF-15 cutoff value of >2 ng/mL was 2.400 (95% CI: 1.854 to 3.106, *p* = 0.000), indicating a significant association between elevated GDF-15 levels and increased mortality rates (Fig. 3C).

### 3.5 Meta-Analysis of Cardiovascular Death

**Fig. 4.**
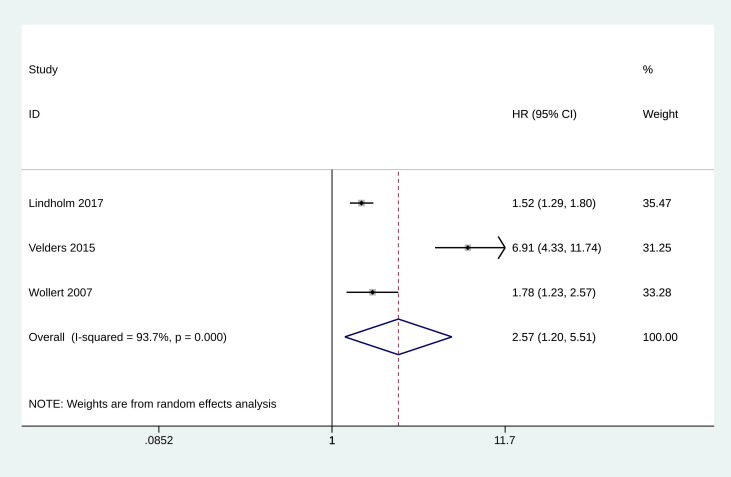
**Forest plot of the incidence of cardiovascular death**.

The analysis for cardiovascular death included data from three studies [13, 14, 26]. The analysis showed significant heterogeneity among the studies (I^2^ = 93.7%, *p* = 0.000), and the random effect model was adopted. The pooled HR for the incidence of cardiovascular death was estimated to be 2.572 (95% CI: 1.200 to 5.510, *p* = 0.015, Fig. 4), indicating a significant association between elevated GDF-15 levels and increased risk of cardiovascular death.

### 3.6 Meta-Analysis of Postoperative AF

**Fig. 5.**
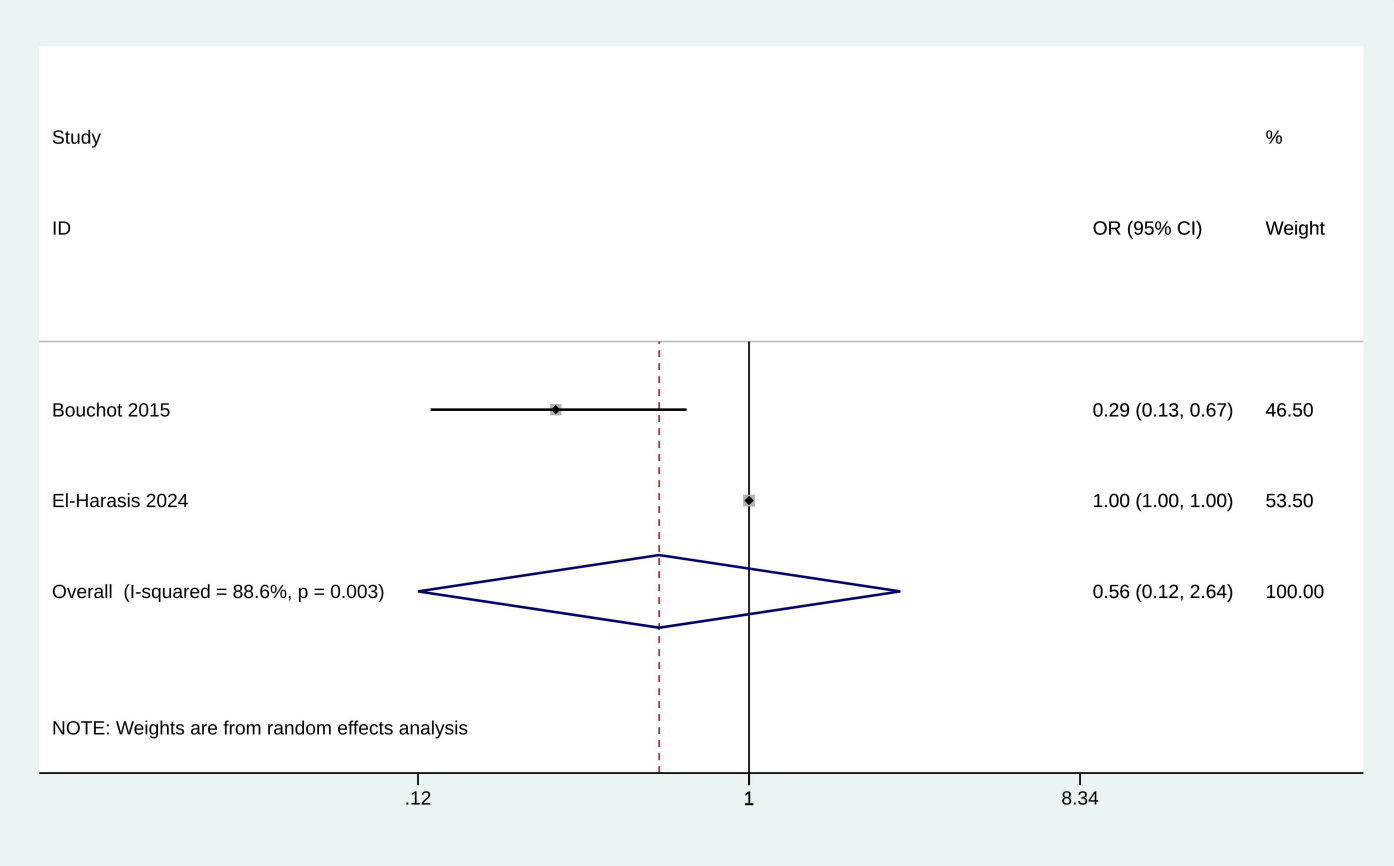
**Forest plot of the incidence of atrial fibrillation**.

The meta-analysis analyzed the association between atrial fibrillation and GDF-15 levels in patients undergoing cardiac surgery. The pooled OR for the incidence of postoperative AF was estimated to be 0.562 (95% CI: 0.120 to 2.637, *p* = 0.465, Fig. 5), indicating no significant difference in the incidence of postoperative AF between high GDF-15 levels and control groups.

### 3.7 Meta-Analysis of AKI

**Fig. 6.**
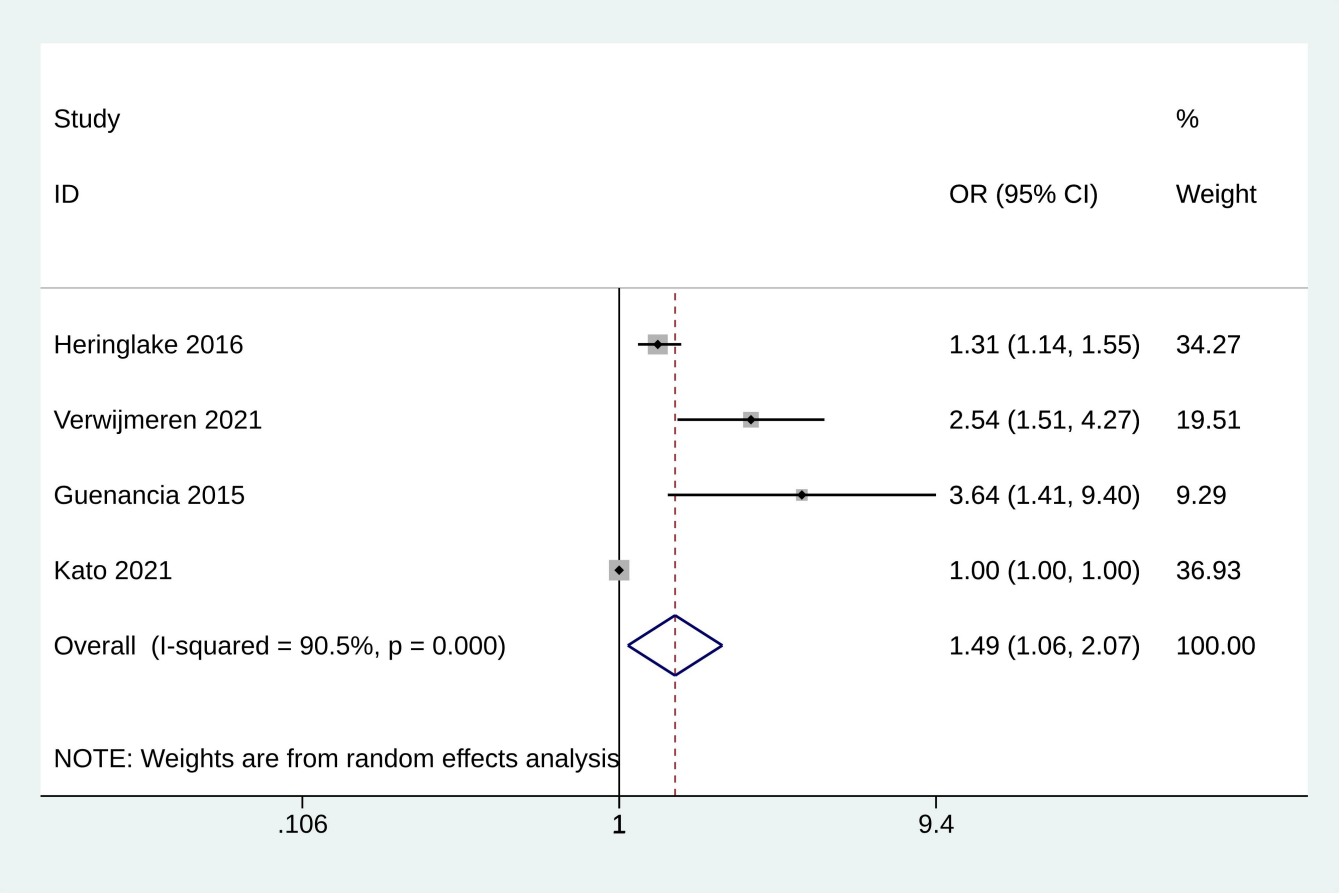
**Forest plot of the incidence of acute kidney injury**.

The analysis for AKI included data from four studies [11, 12, 21, 22]. The pooled OR for the incidence of AKI was estimated to be 1.485 (95% CI: 1.063 to 2.075, *p *= 0.020, Fig. 6), indicating a significant association between elevated GDF-15 levels and an increased risk of AKI.

### 3.8 Meta-Analysis of Spontaneous MI

**Fig. 7.**
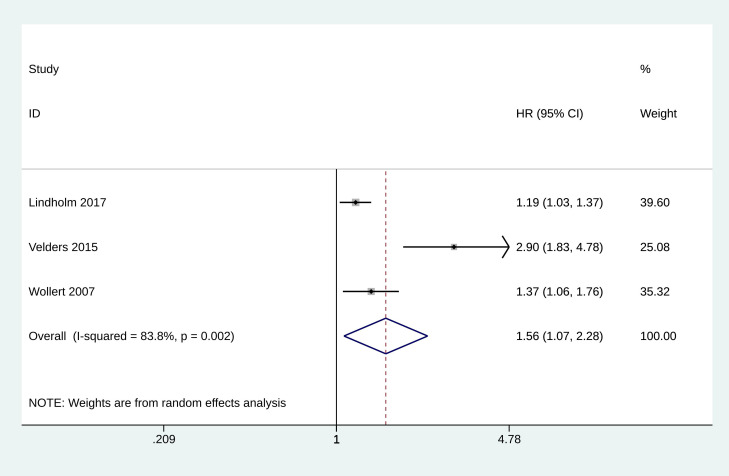
**Forest plot of the incidence of spontaneous myocardial infarction**.

Three studies reported the predictive effect of GDF-15 on spontaneous MI [13, 14, 26]. The pooled HR for the incidence of spontaneous MI was estimated to be 1.564 (95% CI: 1.070 to 2.284, *p* = 0.021, Fig. 7), indicating a significant association between elevated GDF-15 levels and an increased risk of spontaneous MI.

### 3.9 Publication Bias

The funnel plot exhibited an asymmetrical distribution of dots around the pooled effect size, indicating potential publication bias (**Supplementary Fig. 2**). The Egger’s test indicated a significant bias with a coefficient of 3.296503 (*p* = 0.001), suggesting the presence of publication bias. The trim-and-fill analysis showed a consistent pooled effect size before and after adjustment.

## 4. Discussion

This meta-analysis highlights the significant association between elevated GDF-15 levels and various adverse outcomes in patients undergoing cardiac surgery. Elevated GDF-15 levels were significantly linked to increased all-cause mortality, with a more pronounced effect observed in retrospective studies compared to prospective ones. Higher GDF-15 cutoff levels showed a stronger association with increased mortality, indicating that as the cutoff level for GDF-15 increases, the disparity in outcomes between patients with high and low GDF-15 levels becomes more pronounced. Additionally, GDF-15 levels were associated with increased risks of cardiovascular death, acute kidney injury, and spontaneous MI, but not with atrial fibrillation. The analysis of overall adverse outcomes showed no significant difference, but subgroup analyses suggested significant associations primarily in studies with higher GDF-15 cutoff levels. These findings underscore the critical role of GDF-15 as a biomarker for predicting adverse outcomes in cardiovascular interventions patients, emphasizing the need for careful monitoring and management of patients with high GDF-15 levels to improve surgical outcomes.

Postoperative AF was a common complication, and its occurrence ranged from 20% to 45% in those who have undergone CABG surgery [10, 27]. The presence of postoperative AF adversely affected patient outcomes by increasing the duration of hospitalization, escalating healthcare costs, and heightening the risks of stroke, and mortality [28]. Bouchot *et al*. [10] analyzed the postoperative AF in a total of 100 patients undergoing CABG, and demonstrated that low plasma GDF-15 levels before CABG surgery are a strong independent predictor of postoperative AF. However, El-Harasis *et al*. [20] suggested that the inclusion of GDF-15 and other biomarkers did not enhance the predictive ability for the occurrence of AF (*p *= 0.09). This meta-analysis pooled data from these two studies and found that GDF-15 levels were not associated with an increased risk of postoperative AF following cardiovascular interventions. The limited number of studies included in this analysis highlights the need for further research. The current evidence is insufficient to draw definitive conclusions about the predictive value of GDF-15 for postoperative AF. Future studies with larger sample sizes and more rigorous designs are necessary to fully understand the relationship between GDF-15 levels and postoperative AF risk.

AKI represents a common and serious complication following cardiovascular interventions and is linked with adverse outcomes, including prolonged hospital stays, increased healthcare costs, and higher mortality rates [21]. Guenancia *et al*. [21] demonstrated that pre-operative GDF-15 plasma levels are significantly associated with post-operative AKI in CABG patients. The predictive value of pre-operative GDF-15 for AKI was assessed against other biomarkers using receiver operating characteristic (ROC) curves. GDF-15 achieved an area under the curve (AUC) of 0.83, establishing itself as the most effective pre-operative biomarker for predicting AKI. This performance outperformed both the estimated glomerular filtration rate (eGFR) with an AUC of 0.67 and NT-proBNP with an AUC of 0.62 [21]. This meta-analysis pooled data from four studies, further indicates that elevated pre-operative GDF-15 plasma levels are strongly linked to post-operative AKI. These findings reinforce the potential application of GDF-15 as a reliable biomarker for identifying patients at high risk of developing AKI following cardiovascular interventions. Lindholm *et al*. [13] demonstrated that in revascularized patients with non–ST-elevation acute coronary syndrome, adding the extent of NT-proBNP and GDF-15 to clinical risk variables significantly improves the prediction of spontaneous MI. GDF-15, in particular, contributes to better risk stratification, highlighting its potential utility in guiding more intensive or prolonged antithrombotic treatment in this patient population [13]. This meta-analysis further indicates that elevated GDF-15 plasma levels are strongly associated with post-operative spontaneous MI and cardiovascular death in patients with cardiovascular interventions. The findings indicate that GDF-15 enhances risk stratification and may be valuable for predicting these adverse outcomes.

Higher GDF-15 cutoff levels were associated with increased mortality, reflecting a greater disparity in outcomes between patients with high and low GDF-15 levels. While the overall analysis of adverse outcomes did not show a significant difference, subgroup analyses suggested significant associations primarily in studies with higher GDF-15 cutoff levels. Higher cutoff values for GDF-15 displayed more pronounced differences between groups, likely due to increased specificity. However, this increase in specificity may come at the cost of reduced sensitivity, potentially missing some high-risk patients. Therefore, further research is necessary to determine the optimal cutoff value through Youden index. This approach will help balance sensitivity and specificity, ensuring that GDF-15 remains an effective predictive biomarker for adverse outcomes after cardiovascular interventions.

This study has several limitations to be addressed. First, significant heterogeneity was observed among the included studies, particularly regarding the patients undergoing different cardiovascular interventions and the cutoff values applied. This variability may affect the consistency of the findings and limits the ability to draw definitive conclusions. Second, although efforts were made to include all relevant studies, there is a possibility of publication bias, and this bias could affect the overall results of the meta-analysis. Third, this meta-analysis did not analyze the sensitivity and specificity of GDF-15 as a predictive biomarker for adverse outcomes after cardiovascular interventions due to limited data. Future research should include Youden index to determine optimal cutoff values, balancing sensitivity and specificity, to enhance the clinical utility of GDF-15 in risk stratification. Fourth, this study analyzed GDF-15 as a predictor of all-cause mortality, cardiovascular death, atrial fibrillation, AKI, and spontaneous MI as adverse outcomes [16]. However, it did not assess bleeding events or other adverse outcomes, indicating a limitation that future research should address by further analyzing these factors. Fifth, this study primarily focused on the predictive value of GDF-15 and did not include comparisons with other established biomarkers, such as NT-proBNP, C-reactive protein (CRP), and albumin. This represents a limitation of our research. Future large-scale randomized controlled trials are needed to analyze the advantages of GDF-15 in relation to other prognostic indicators. Finally, it is important to note that 10 of the 13 studies included in this study were published between 2015 and 2019. Changes in treatment approaches for cardiovascular diseases during this time may influence the relevance and applicability of the findings. This temporal limitation should be considered when interpreting the results of this systematic review.

## 5. Conclusion

In conclusion, this systematic review and meta-analysis indicated that GDF-15 is associated with increased risks of all-cause mortality, cardiovascular death, AKI, and spontaneous MI in patients undergoing cardiovascular interventions. Given the heterogeneity observed across the studies, particularly with respect to the diverse surgical techniques employed, it is imperative that the finding be interpreted with caution. Further research is required to elucidate the underlying mechanisms of these associations and to refine the clinical application of GDF-15 as a prognostic biomarker.

## Availability of Data and Materials

All data generated or analyzed during this study are included in this article and supplementary information files.

## Supplementary Material

Supplementary material associated with this article can be found, in the online version, at https://doi.org/10.31083/RCM28279.
