# Supplementary material for: Predictive Effect of GDF-15 on Adverse Outcomes After Cardiovascular Interventions: A Systematic Review and Meta-Analysis
Source: Rev Cardiovasc Med. 2025 Apr 16;26(4):28279. doi: 10.31083/RCM28279 (PMC12059790; doi:10.31083/RCM28279)
Supplement: Supplementary file 1 [file 2153-8174-26-4-28279-s1.zip › Supplementary Material-V2.docx]

Figure S1. Sensitivity analysis of all-cause mortality.


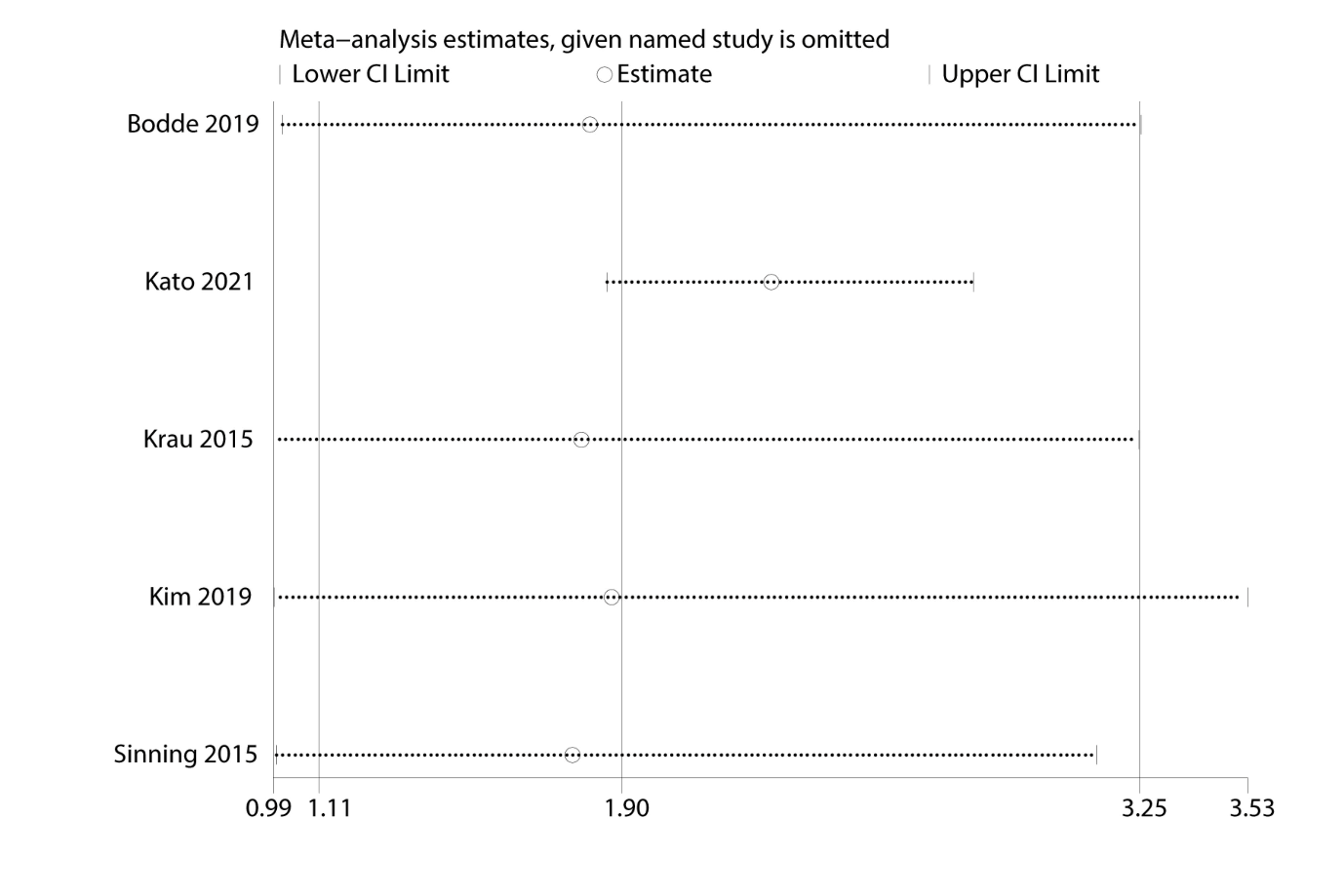


Figure S2. Funnel Plot for publication bias.


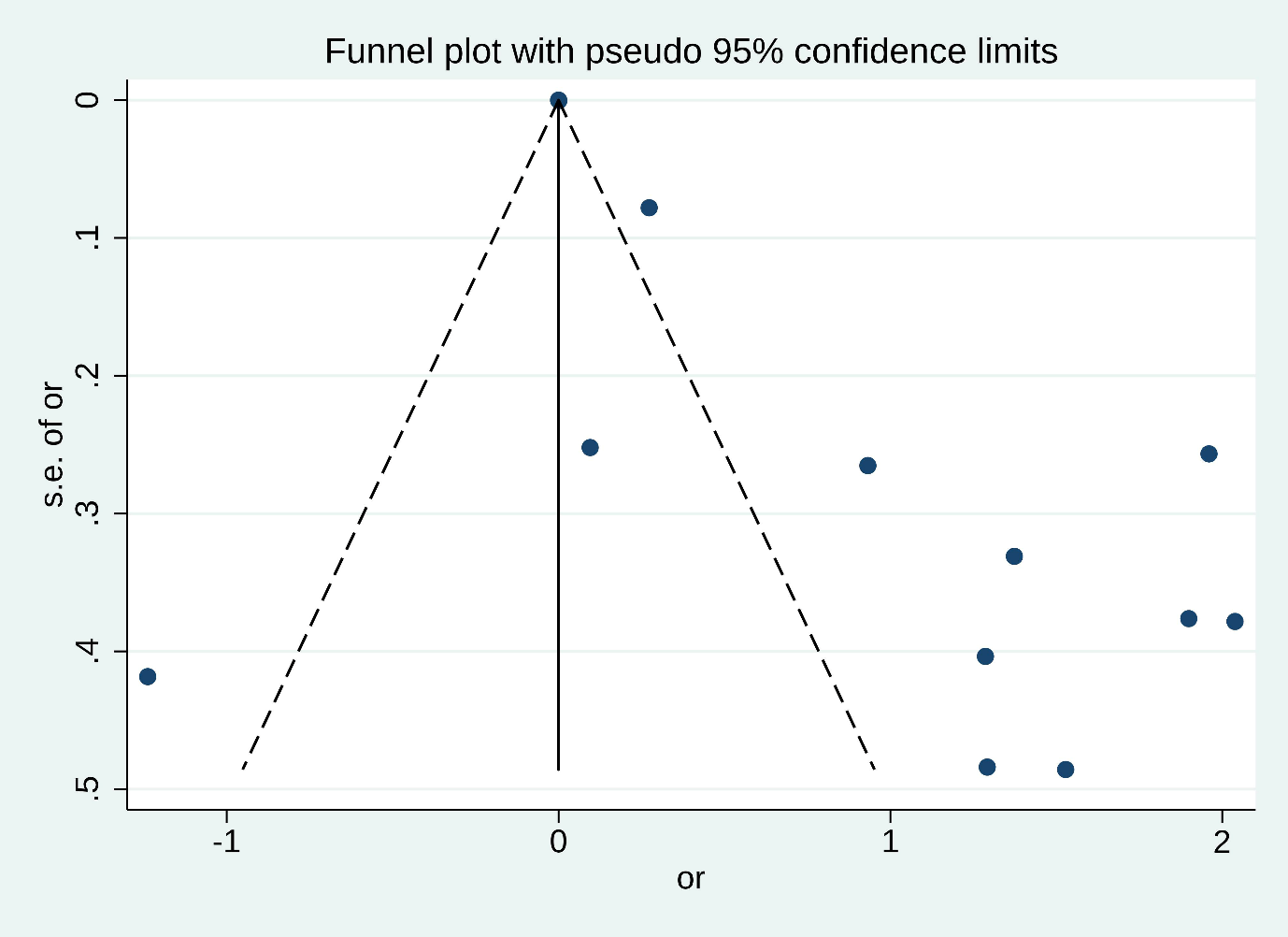


**Table S1. Search strategy.**

**Search strategy of PubMed**

| NO. | Search Details | Results |
| --- | --- | --- |
| #5 | (#1 OR #2) AND (#3 OR #4) | 187 |
| #4 | ((((((((Cardiac Surgical Procedure) OR (Heart Surgical Procedures)) OR (Heart Surgical Procedure)) OR (cardiac surgery)) OR (cardiac surgical procedures)) OR (cardio surgery)) OR (heart operation)) OR (myocardial resection)) OR (heart surgery) | 832,631 |
| #3 | "Cardiac Surgical Procedures"[Mesh] | 249,336 |
| #2 | (((((((((((((((((macrophage inhibitory cytokine-1 protein, human) OR (MIC-1 protein, human)) OR (PLAB protein, human)) OR (non-steroidal anti-inflammatory drug-activated gene-1 protein, human)) OR (prostate-derived factor protein, human)) OR (PTGFB protein, human)) OR (GDF-15 protein, human)) OR (NAG-1 protein, human)) OR (growth differentiation factor 15 protein, human)) OR (placental transforming growth factor beta protein, human)) OR (GDF 15)) OR (GDF15)) OR (macrophage inhibitory cytokine 1)) OR (placental bone morphogenetic protein)) OR (placental TGF beta)) OR (placental transforming growth factor beta)) OR (prostate differentiation factor)) OR (growth differentiation factor 15) | 6229 |
| #1 | "GDF15 protein, human" [Supplementary Concept] | 1066 |

**Search strategy of EMBASE**

| No. | Query | Results |
| --- | --- | --- |
| #5 | (#1 OR #2) AND (#3 OR #4) | 242 |
| #4 | 'cardiac surgical procedure':ti,ab,kw OR 'heart surgical procedures':ti,ab,kw OR 'heart surgical procedure':ti,ab,kw OR 'cardiac surgery':ti,ab,kw OR 'cardiac surgical procedures':ti,ab,kw OR 'cardiosurgery':ti,ab,kw OR 'heart operation':ti,ab,kw OR 'myocardial resection':ti,ab,kw OR 'heart surgery':ti,ab,kw | 101,121 |
| #3 | 'heart surgery'/exp | 483,394 |
| #2 | 'macrophage inhibitory cytokine-1 protein, human':ti,ab,kw OR 'mic-1 protein, human':ti,ab,kw OR 'plab protein, human':ti,ab,kw OR 'non-steroidal anti-inflammatory drug-activated gene-1 protein, human':ti,ab,kw OR 'prostate-derived factor protein, human':ti,ab,kw OR 'ptgfb protein, human':ti,ab,kw OR 'gdf-15 protein, human':ti,ab,kw OR 'nag-1 protein, human':ti,ab,kw OR 'growth differentiation factor 15 protein, human':ti,ab,kw OR 'placental transforming growth factor beta protein, human':ti,ab,kw OR 'gdf 15':ti,ab,kw OR 'gdf15':ti,ab,kw OR 'macrophage inhibitory cytokine 1':ti,ab,kw OR 'placental bone morphogenetic protein':ti,ab,kw OR 'placental tgf beta':ti,ab,kw OR 'placental transforming growth factor beta':ti,ab,kw OR 'prostate differentiation factor':ti,ab,kw OR 'growth differentiation factor 15':ti,ab,kw | 4740 |
| #1 | 'growth differentiation factor 15'/exp | 5731 |

**Search strategy of Cochrane Library**

| NO. | Search deatiles | Hits |
| --- | --- | --- |
| #1 | (macrophage inhibitory cytokine-1 protein, human):ti,ab,kw OR (MIC-1 protein, human):ti,ab,kw OR (PLAB protein, human):ti,ab,kw OR (non-steroidal anti-inflammatory drug-activated gene-1 protein, human):ti,ab,kw OR (prostate-derived factor protein, human):ti,ab,kw OR (PTGFB protein, human):ti,ab,kw OR (GDF-15 protein, human):ti,ab,kw OR (NAG-1 protein, human):ti,ab,kw OR (growth differentiation factor 15 protein, human):ti,ab,kw OR (placental transforming growth factor beta protein, human):ti,ab,kw OR (GDF 15):ti,ab,kw OR (GDF15):ti,ab,kw OR (macrophage inhibitory cytokine 1):ti,ab,kw OR (placental bone morphogenetic protein):ti,ab,kw OR (placental TGF beta):ti,ab,kw OR (placental transforming growth factor beta):ti,ab,kw OR (prostate differentiation factor):ti,ab,kw OR (growth differentiation factor 15):ti,ab,kw | 541 |
| #2 | MeSH descriptor: [Cardiac Surgical Procedures] explode all trees | 17,280 |
| #3 | (Cardiac Surgical Procedure):ti,ab,kw OR (Heart Surgical Procedures):ti,ab,kw OR (Heart Surgical Procedure):ti,ab,kw OR (cardiac surgery):ti,ab,kw OR (cardiac surgical procedures):ti,ab,kw OR (cardiosurgery):ti,ab,kw OR (heart operation):ti,ab,kw OR (myocardial resection):ti,ab,kw OR (heart surgery):ti,ab,kw | 49,214 |
| #4 | #1 and (#2 or #3) | 25 |

**Search strategy of Web of science**

| NO. | Search deatiles | Hits |
| --- | --- | --- |
| #1 | ((((((((((((((((TS=(macrophage inhibitory cytokine-1 protein, human) OR TS=(MIC-1 protein, human)) OR TS=(PLAB protein, human)) OR TS=(non-steroidal anti-inflammatory drug-activated gene-1 protein, human)) OR TS=(prostate-derived factor protein, human)) OR TS=(PTGFB protein, human)) OR TS=(GDF-15 protein, human)) OR TS=(NAG-1 protein, human)) OR TS=(growth differentiation factor 15 protein, human)) OR TS=(placental transforming growth factor beta protein, human)) OR TS=(GDF 15)) OR TS=(GDF15)) OR TS=(macrophage inhibitory cytokine 1)) OR TS=(placental bone morphogenetic protein)) OR TS=(placental TGF beta)) OR TS=(placental transforming growth factor beta)) OR TS=(prostate differentiation factor)) OR TS=(growth differentiation factor 15) | 17,811 |
| #2 | (((((((TS=(Cardiac Surgical Procedure) OR TS=(Heart Surgical Procedures)) OR TS=(Heart Surgical Procedure)) OR TS=(cardiac surgery)) OR TS=(cardiac surgical procedures)) OR TS=(cardiosurgery)) OR TS=(heart operation)) OR TS=(myocardial resection)) OR TS=(heart surgery) | 186,822 |
| #3 | #2 AND #1 | 67 |

**Table S2. Newcastle-Ottawa quality assessment of the included studies.**

| Author year | Definition adequate | Representativeness of the cases | Selection of controls | Definition of controls | Comparability of cases and controls on the basis of the design or analysis | Ascertainment of exposure | The same method of ascertainment for cases and controls | Nonresponse rate | Total quality scores |
| --- | --- | --- | --- | --- | --- | --- | --- | --- | --- |
| Heringlake 2016 | 1 | 1 | 1 | 2 | 1 | 1 | 1 | 1 | 9 |
| Bodde 2019 | 1 | 1 | 1 | 2 | 1 | 1 | 1 | 1 | 9 |
| Verwijmeren 2021 | 1 | 1 | 1 | 0 | 1 | 1 | 1 | 1 | 7 |
| Bouchot 2015 | 1 | 1 | 1 | 1 | 1 | 1 | 1 | 1 | 8 |
| Lindholm 2017 | 1 | 1 | 1 | 2 | 1 | 1 | 1 | 1 | 9 |
| El-Harasis 2024 | 1 | 1 | 1 | 1 | 1 | 1 | 1 | 1 | 8 |
| Guenancia 2015 | 1 | 1 | 1 | 1 | 1 | 1 | 1 | 1 | 8 |
| Kato 2021 | 1 | 1 | 1 | 2 | 1 | 1 | 1 | 1 | 9 |
| Krau 2015 | 1 | 1 | 1 | 2 | 1 | 1 | 1 | 1 | 9 |
| Kim 2019 | 1 | 1 | 1 | 2 | 1 | 1 | 1 | 1 | 9 |
| Sinning 2015 | 1 | 1 | 1 | 2 | 1 | 1 | 1 | 1 | 9 |
| Velders 2015 | 1 | 1 | 1 | 2 | 1 | 1 | 1 | 1 | 9 |
| Wollert 2007 | 1 | 1 | 1 | 2 | 1 | 1 | 1 | 1 | 9 |
